# Associations Between Different Eosinophil Activity Markers and Clinical Outcomes in Young Asthmatics

**DOI:** 10.1002/clt2.70167

**Published:** 2026-03-31

**Authors:** Nils Oskar Jõgi, Nikolaos Tsolakis, Anders Sjölander, Robert Movérare, Christer Janson, Andrei Malinovschi, Kjell Alving

**Affiliations:** ^1^ Department of Medical Sciences, Clinical Physiology Uppsala University Uppsala Sweden; ^2^ Department of Women's and Children's Health Uppsala University Uppsala Sweden; ^3^ Thermo Fisher Scientific Uppsala Sweden; ^4^ Department of Medical Sciences, Respiratory‐, Allergy‐ and Sleep Research Uppsala University Uppsala Sweden

**Keywords:** asthma, blood eosinophils, ECP, EDN, FeNO

## Abstract

**Background:**

Identifying biomarkers for different aspects of asthma morbidity is crucial for effective disease monitoring and management. The performance of fractional exhaled nitric oxide (FeNO), blood eosinophil count (BEC), and eosinophil activity markers should be evaluated.

**Objective:**

This study aimed to compare the associations of FeNO, BEC, serum and plasma eosinophil‐derived neurotoxin (S‐ and P‐EDN), and serum eosinophilic cationic protein (S‐ECP) with important clinical outcomes in asthma.

**Methods:**

In total, 390 individuals aged 10–35 years with physician‐diagnosed asthma were included. Asthma control test (ACT), spirometry, and methacholine challenge tests were used to assess asthma morbidity. The biomarkers' associations with asthma outcomes: impaired spirometry (FEV_1_ < 80% predicted, FEV_1_/FVC < lower limit of normal), airway hyperresponsiveness (AHR), uncontrolled asthma (ACT < 20) were analysed with multiple logistic regression models adjusted for body mass index, sex, age, immunoglobulin E sensitisation, smoking, and controller medication. The Akaike information criterion (AIC) was calculated for each model, to compare biomarker performance.

**Results:**

S‐EDN had the lowest AIC value of all biomarkers in models assessing the association with reduced lung function, showing better model fit than P‐EDN and S‐ECP. However, FeNO and BEC had the lowest AIC values for AHR. FeNO was the only biomarker associated with uncontrolled asthma.

**Conclusion:**

S‐EDN may serve as an alternative to BEC in research setting and could be relevant in assessing airway obstruction. However, adding S‐EDN as a routine biomarker alongside FeNO and BEC may offer limited additional clinical value. Further research in larger cohorts is needed to validate these findings and establish optimal cut‐offs.

**Abbreviations**
ACT: asthma control test
AHR: airway hyperresponsiveness
AIC: Akaike information criterion
aOR: adjusted odds ratio
ATS: American Thoracic Society
BEC: blood eosinophil count
BMI: body mass index
ECP: eosinophil cationic protein
EDN: eosinophil‐derived neurotoxin
EDTA: ethylenediaminetetraacetic acid
FeNO: fractional exhaled nitric oxide
FVC: forced vital capacity
GAGs: glycosaminoglycans
GLI: global lung initiative
ICS: inhaled corticosteroid
IgE: immunoglobulin E
LABA: long‐acting beta‐2 agonist
LLN: lower limit of normal
LTRAs: leukotriene‐receptor antagonists
MIDAS: Minimally‐Invasive Diagnostic procedures in allergy, Asthma, or food hypersensitivity Study
OR: odds ratio
P‐EDN: plasma eosinophil‐derived neurotoxin
PD_20_
: provocative dose causing a 20% fall in FEV_1_
S‐ECP: serum eosinophil cationic protein
S‐EDN: serum eosinophil‐derived neurotoxin
ULN: upper limit of normal

## 1 Introduction

Asthma is a chronic multifactorial disease affecting all age groups and poses a substantial global health burden [1, 2]. Airway obstruction, characterised by variable airflow limitation and reduced lung function, is a hallmark feature of asthma, contributing significantly to its morbidity and mortality. Identifying biomarkers that associate with the degree of airway obstruction holds importance in asthma management. In particular, identification of prognostic biomarkers could enable a timely intervention, to potentially limit disease progression [3]. Among the array of potential biomarkers, blood eosinophil count (BEC) and fractional exhaled nitric oxide (FeNO) have emerged as clinically useful biomarkers, in both asthma diagnosis and management [4, 5, 6, 7]. However, there can be a substantial discordance in the information provided between sputum eosinophils count, BEC, and FeNO [8]. Thus, there is an interest in alternatives, such as markers of eosinophil activation.

Serum eosinophil cationic protein (S‐ECP), which is released during eosinophil degranulation [9, 10], emerged as a promising biomarker for eosinophil activation and disease severity during the 1990s [11, 12, 13]. For example, it has been reported to be a reliable biomarker in the diagnosis of childhood asthma, with additional value in combination with FeNO and spirometry [14]. S‐ECP has also shown the potential to be a substitute marker for BEC [14]. Furthermore, S‐ECP seems to be useful in assessing the effectiveness of ordinary and biological asthma treatment [15, 16, 17], as well as in asthma risk assessment [18].

However, S‐ECP measurement has some limitations. ECP is primarily released in vitro during the coagulation phase [19], and the levels are therefore dependent on the time of coagulation and the temperature during serum preparation [20, 21, 22]. This necessitates strict sample handling procedures to avoid variations during the coagulation phase. Additionally, ECP is a very basic granule protein with high affinity for negatively charged glycosaminoglycans (GAGs) [23] and will mostly be bound to cells and the endothelium, and is detected only in very low amounts in plasma. Serum eosinophil‐derived neurotoxin (S‐EDN), another product of eosinophil activation and degranulation, has demonstrated promising potential as a marker for eosinophilic inflammation [3, 24], exhibiting close associations with airway inflammation [25] and asthma severity [26]. Recent studies have shown that S‐EDN can be a better marker of asthma control than BEC [27], and elevated S‐EDN levels at 1 and 3 years of age have been shown to associate with asthma and allergic disease [28]. Moreover, EDN is less cationic than ECP and thus sticks less to GAGs and plastic surfaces [19]; it also shows limited circadian variation in serum [29]. Therefore, S‐EDN, and possibly P‐EDN, may have advantages over S‐ECP and BEC in the assessment of patients with asthma. However, combining different biomarkers could possibly better reflect asthma symptoms [30] and morbidity [31], and identify those at risk of asthma exacerbations [32, 33, 34].

Whereas BEC and FeNO have been extensively studied in asthma, the potential role of S‐EDN in assessing asthma morbidity remains relatively unexplored. To the best of our knowledge, this is the first study to directly compare BEC, S‐EDN, S‐ECP, P‐EDN, and FeNO together against lung function, AHR, and asthma control in the same cohort of adolescents and young adults. Thus, the aim of this study was to evaluate the associations of S‐EDN, P‐EDN, and S‐ECP with lung function, airway hyperresponsiveness, and disease control in young patients with asthma, and compare them with BEC and FeNO.

## 2 Methods

### 2.1 Study Population

**FIGURE 1:**
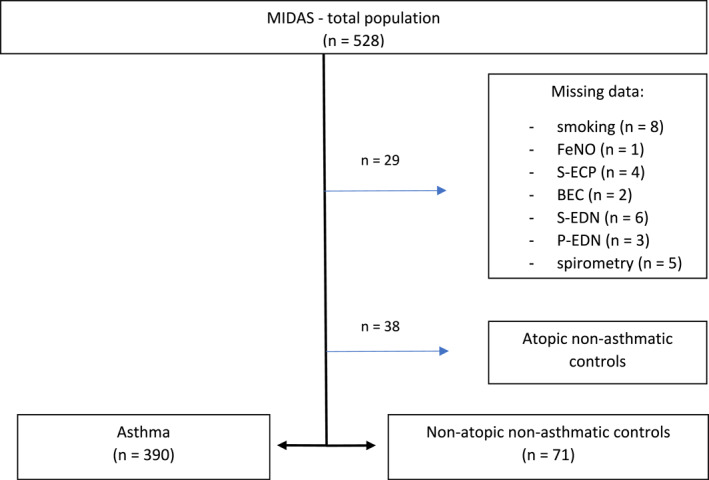
Flowchart of study population selection.

This cross‐sectional, single‐centre study was based on subjects who participated in the Minimally‐Invasive Diagnostic procedures in allergy, Asthma, or food hypersensitivity Study (MIDAS). Subjects were recruited from both primary and secondary care in Uppsala, Sweden. A total of 390 subjects aged 10–35 years with physician‐diagnosed asthma and daily treatment with an inhaled corticosteroid (ICS), or a leukotriene‐receptor antagonist (LTRA), or both during at least 3 of the preceding 12 months were included in the present study [35, 36]. Exclusion criteria included acute and other chronic respiratory diseases, active tuberculosis, and recorded blood‐borne disease. Additionally, 71 non‐atopic, non‐asthmatic controls (age 10–35 years), randomly chosen from the population registry, with available clinical data, were included in the study to establish population‐based cut‐off values for EDN and ECP. The flow chart for study population selection can be seen in Figure 1.

### 2.2 Clinical Asthma Characteristics

Subjects responded to questions regarding asthma symptoms in the preceding 12 months [37]. The degree of asthma control was assessed using the asthma control test (ACT) [38]. Information on asthma attacks was self‐reported. Each subject's use of ICS and LTRA was recorded through an interview. Information on the prescribed daily doses of ICS was collected from medical records.

### 2.3 Exhaled Nitric Oxide

FeNO values were measured in accordance with current American Thoracic Society (ATS)/European Respiratory Society recommendations using a chemiluminescence analyser at a flow rate of 50 mL/s (NIOX Flex; Aerocrine AB, Solna, Sweden) [39]. FeNO levels were regarded as normal if they were below 20 ppb for a patient younger than 12 years or below 25 ppb for a Patient 12 years or older, whereas values of 20 ppb or more and 25 ppb or more, respectively, were considered increased [40].

### 2.4 Lung Function

Flow‐volume curves were obtained in accordance with the ATS recommendations with a Masterscope spirometer (Jaeger Master, Wurzberg, Germany). Impaired lung function values were defined as either forced expiratory volume during first second (FEV_1_) < 80% predicted or FEV_1_/forced vital capacity (FVC) ratio < lower limit of normal (LLN), all calculated according to Global Lung Initiative (GLI) reference values [41].

### 2.5 Airway Hyperresponsiveness

Methacholine provocation was performed with the Aerosol Provocation System (Viasys Healthcare GmbH, Hoechberg, Germany) using a simplified protocol described in detail elsewhere [35]. Airway responsiveness was defined as normal when the methacholine cumulative dose causing a 20% decrease in FEV_1_ (PD_20_) was > 1.0 mg. Airway hyperresponsiveness (AHR) was defined as borderline to mild at 0.3–1.0 mg and moderate to severe at < 0.3 mg, in accordance with the method of Schulze et al. [42].

### 2.6 Blood Eosinophils

Venous blood (EDTA) was drawn and BEC was measured at the Department of Clinical Chemistry and Pharmacology at Uppsala University Hospital using a routine method (Cell‐Dyn 4000; Abbott, Abbott Park, IL). Subjects were divided into two groups based on BEC: normal (< 0.3 × 10^9^/L) or increased (≥ 0.3 × 10^9^/L) [43].

### 2.7 Atopy

Immunoglobulin E (IgE) antibodies to common aeroallergens and food allergens were measured with Phadiatop and the fx5 test, respectively (ImmunoCAP Specific IgE assay; Phadia AB/Thermo Fisher Scientific, Uppsala, Sweden) [44]. Subjects were defined as atopic if they had IgE‐antibody concentrations of 0.35 kU_A_/L or greater.

### 2.8 Analysis of Eosinophil Activity Markers

S‐ECP, S‐EDN, and P‐EDN (EDTA) were measured using the ImmunoCAP ECP assay and ImmunoCAP EDN assay (Phadia AB/Thermo Fisher Scientific). The latter assay is for research use only and has been described elsewhere [45]. Blood was drawn into EDTA tubes for plasma preparation, and into gel tubes for serum preparation (coagulation was allowed for 60–120 min at 21°C–23°C). Centrifugation was done at 1000–1300 × *g* at +4°C, and plasma/serum was immediately aliquoted and frozen at −80°C.

### 2.9 Statistics

Pearson's chi‐square tests were used for categorical values and *t*‐tests for log‐transformed biomarkers, to evaluate the difference between asthmatics and controls. Pearson's correlation matrix was used to assess the correlations between the different biomarkers.

The upper limits of normal (ULN) for S‐ECP, S‐EDN, and P‐EDN were defined as the 95th percentile of values for the non‐atopic controls.

Univariate and multiple logistic regression models with asthma outcome variables (abnormal lung function, moderate‐to‐severe AHR, and uncontrolled asthma) were used to estimate the odds ratios of these events in relation to elevated FeNO, BEC, S‐ECP, S‐EDN, and P‐EDN. All models were adjusted for the confounders body mass index (BMI) group, sex, age, IgE sensitisation, smoking, ICS dose (calculated as budesonide equivalents and included as a continuous variable), and use of LTRA. BMI groups were calculated based on percentiles for subjects < 18 years old (< 5th percentile underweight, 5–85th normal, 85–95th overweight, > 95th obese). For adults, the World Health Organization cut‐offs were used: < 18.5 kg/m^2^ underweight, 18.5–24.9 kg/m^2^ normal weight, 25–29.9 kg/m^2^ overweight, and > 30 kg/m^2^ obese.

Akaike information criterion (AIC) values were calculated for each biomarker and model in order to compare the biomarkers with each other. Lower AIC values indicate better model fit, reflecting a stronger association between the biomarker and the outcome. We used the formula Δ*i* = AIC_*i*_ − AIC_min_, where Δ*i* is the relative difference between the best model and other models in the set. Models with Δ*i* ≤ 2 have substantial support (evidence), those with Δ*i* 4–7 have considerably less support, and models with Δ*i* > 10 have essentially no support [46].

## 3 Results

Participant characteristics are given in Table 1. Uncontrolled asthma (ACT score < 20) was found in 30.5%, whereas 38 (9.7%) subjects had ACT scores 5–15 and 85 (20.8%) had ACT scores 16–19. Based on the 95th percentile of the non‐atopic healthy controls, the cut‐offs for increased S‐ECP, S‐EDN, and P‐EDN were considered to be 25.7, 62.0, and 20.7 μg/L, respectively.

**TABLE 1:** Population characteristics.

	Asthmatics (*n* = 390)	Non‐atopic non‐asthmatic (*n* = 71)	*p*‐value of difference between groups
Female (%)	52.1	60.6	0.19
Age (median; range)	19.2 (10.0–35.8)	17.9 (10.6–32.5)	0.94
Height (median; range)	167.0 (130–197)	169.0 (142–198)	< 0.05
Normal weight (%)	63.1	71.8	0.37
Overweight (%)	19.0	16.9	
Obese (%)	14.1	7.0	
Underweight (%)	3.9	4.2	
Current smoking (%)	4.6	12.7	< 0.01
Ex‐smoker (%)	19.2	28.2	
Never smoker (%)	76.2	59.2	
ICS dose (last 3 months) (mean; range)	379 mg/day (0–2000)	0	
ICS/LABA use last 3 months (%)	98.0	0	
LTRA use last 3 months (%)	19.7	0	
Atopy (%)	79.7	0	
FeNO (ppb) (mean; ± SD)	21.5 (± 21.3)	13.5 (± 12.2)	< 0.01
FeNO high^a^ (%)	26.7	9.9	< 0.01
BEC (per μL) (mean; ± SD)	264 (± 251)	145 (± 149)	< 0.01
BEC high (> 300 per μL)	37.2	14.1	< 0.01
S‐EDN (μg/L) (mean; ±SD)	51.8 (± 44.3)	30.0 (± 21.4)	< 0.01
S‐EDN high (> 62 μg/L) (%)	27.4	5.6	< 0.01
P‐EDN (μg/L) (mean; ± SD)	18.1 (± 12.1)	12.5 (± 6.4)	< 0.01
P‐EDN high (> 20.7 μg/L) (%)	28.5	4.2	< 0.01
S‐ECP (μg/L) (mean; ± SD)	16.9 (± 14.5)	10.9 (± 10.9)	< 0.01
S‐ECP high (> 25.7 μg/L) (%)	15.0	4.2	0.20
FEV_1_ (% predicted) (mean; ± SD)	94.6 (± 14.4)	98.8 (± 14.4)	< 0.01
FEV_1_ < 80% predicted (%)	13.9	7.0	0.10
FEV_1_/FVC ratio (mean; ± SD)	0.81 (± 0.08)	0.86 (± 0.07)	< 0.01
FEV_1_/FVC ratio < LLN (%)	20.5	5.6	< 0.01
PD_20_ (mg) (mean; ± SD)	(*n* = 355)	(*n* = 68)	< 0.01
	1.80 (± 2.76)	3.12 (± 3.31)	
Moderate/severe AHR (PD_20_ < 0.3 mg) (%)	51.8	26.5	< 0.01
ACT score (mean; ± SD)	20.4 (± 3.4)	NA	
Uncontrolled asthma (ACT score < 20) (%)	30.5	NA	
≥ 2 asthma attacks within 3 months prior to the study (%)	33.9	NA	

Abbreviation: LABA, long‐acting beta‐2 agonist.

^a^ Less than 12 years of age: > 20 ppb, 12 years of age or older: > 25 ppb.

When evaluating each biomarker in relation to asthma outcomes, both elevated BEC and elevated S‐EDN were associated with an increased odds ratio (OR) of having FEV_1_ < 80% predicted (Table 2). The other markers did not significantly associate with FEV_1_. All biomarkers except S‐ECP were associated with increased odds of having an FEV_1_/FVC ratio under the LLN. Furthermore, all five biomarkers were associated with moderate to severe AHR, with FeNO showing the strongest association and P‐EDN the weakest. FeNO was the only biomarker associated with uncontrolled asthma (Table 2).

**TABLE 2:** Adjusted ORs^a^ for having airway obstruction if having increased levels of different inflammatory markers in a logistic regression model.

Marker	Participants with increased values (*n*)	FEV_1_ < 80% predicted (aOR; 95% CI)	FEV_1_/FVC ratio < LLN (%) (aOR; 95% CI)	Moderate/severe AHR (PD_20_ < 0.3 mg) (aOR; 95% CI)	Uncontrolled asthma (ACT score < 20) (aOR; 95% CI)
BEC	145	**1.9 (1.0–3.6)**	**1.8 (1.0–3.0)**	**4.3 (2.5–7.4)**	1.2 (0.7–1.9)
FeNO	104	1.7 (0.9–3.3)	**1.8 (1.0–3.1)**	**5.0 (2.7–9.1)**	**2.0 (1.2–3.3)**
S‐EDN	107	**2.3 (1.2–4.5)**	**2.6 (1.5–4.4)**	**3.3 (1.9–5.9)**	1.0 (0.6–1.7)
P‐EDN	111	1.6 (0.9–3.2)	**2.1 (1.2–3.6)**	**2.3 (1.3–4.0)**	0.8 (0.5–1.4)
S‐ECP	66	1.4 (0.7–3.1)	1.0 (0.5–2.0)	**2.6 (1.4–5.0)**	0.97 (0.53–1.80)

*Note:* Bold text marks the associations that were statistically significant (95% confidence interval [CI] does not include 1).

^a^ Adjusted for BMI group, sex, age, atopy, ICS dose, LTRA usage, and smoking.

**FIGURE 2:**
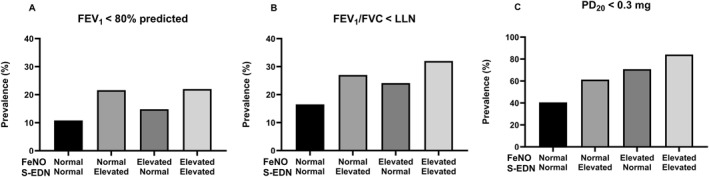
FeNO (< 25 ppb vs. ≥ 25 ppb for adults; < 20 ppb vs. ≥ 20 ppb for children < 12 years) and S‐EDN (< 62 μg/L vs. ≥ 62 μg/L) in relation to the prevalence of airway obstruction (defined as FEV_1_ < 80% of predicted [A] or FEV_1_/FVC ratio < LLN [B]) and moderate to severe AHR (PD_20_ < 0.3 mg [C]).

When combining biomarkers, elevated S‐EDN and elevated FeNO had a strengthened association with asthma outcomes (Table 3, Figure 2).

**TABLE 3:** Adjusted ORs^a^ for having airway obstruction if having increased FeNO and S‐EDN levels in a logistic regression model.

FeNO level	S‐EDN	No	FEV_1_ < 80% predicted (aOR; 95% CI)	FEV_1_/FVC ratio < LLN (%) (aOR; 95% CI)	Moderate/severe AHR (PD_20_ < 0.3 mg) (aOR; 95% CI)	Uncontrolled asthma (ACT score < 20) (aOR; 95% CI)
Normal	Normal	237	1	1	1	1
Normal	Increased	49	1.8 (0.7–4.5)	**2.6 (1.2–5.5)**	**2.6 (1.2–5.5)**	0.9 (0.4–1.9)
Increased	Normal	46	1.1 (0.4–3.1)	1.6 (0.7–3.6)	**4.6 (2.0–10.2)**	**2.5 (1.2–5.1)**
Increased	Increased	58	**3.0 (1.3–6.7)**	**3.0 (1.5–6.0)**	**7.9 (3.4–18.6)**	1.6 (0.8–3.0)

*Note:* Bold text marks the associations that were statistically significant (95% confidence interval [CI] does not include 1).

^a^ Adjusted for BMI group, sex, age, atopy, ICS dose, LTRA usage, and smoking.

Using a combined score of elevated S‐EDN, elevated FeNO, and/or elevated BEC resulted in stronger associations with all asthma outcomes except asthma control. For example, an elevated level of all three biomarkers resulted in four times higher adjusted OR (aOR) than any single elevated biomarker (Table 4). A combined score of all five biomarkers showed no further strengthening of associations with the asthma outcomes (data not shown).

**TABLE 4:** Adjusted ORs^a^ for having airway obstruction if having increased BEC, FeNO, and/or S‐EDN levels in a logistic regression model.

No of positive markers (BEC, FeNO, S‐EDN)	No	FEV_1_ < 80% predicted (aOR; 95% CI)	FEV_1_/FVC ratio < LLN (%) (aOR; 95% CI)	Moderate/severe AHR (PD_20_ < 0.3 mg) (aOR; 95% CI)	Uncontrolled asthma (ACT score < 20) (aOR; 95% CI)
0	203	1	1	1	1
1	72	0.9 (0.3–2.1)	0.9 (0.4–1.9)	**2.4 (1.2–4.6)**	1.0 (0.5–2.0)
2	61	1.6 (0.7–3.8)	**2.7 (1.3–5.4)**	**5.3 (2.5–11.3)**	1.2 (0.6–2.3)
3	54	**3.2 (1.4–7.4)**	**2.5 (1.2–5.3)**	**9.5 (3.8–23.8)**	1.7 (0.8–3.3)

*Note:* Bold text marks the associations that were statistically significant (95% confidence interval [CI] does not include 1).

^a^ Adjusted for BMI category, sex, age, atopy, ICS dose, LTRA usage and smoking.

**FIGURE 3:**
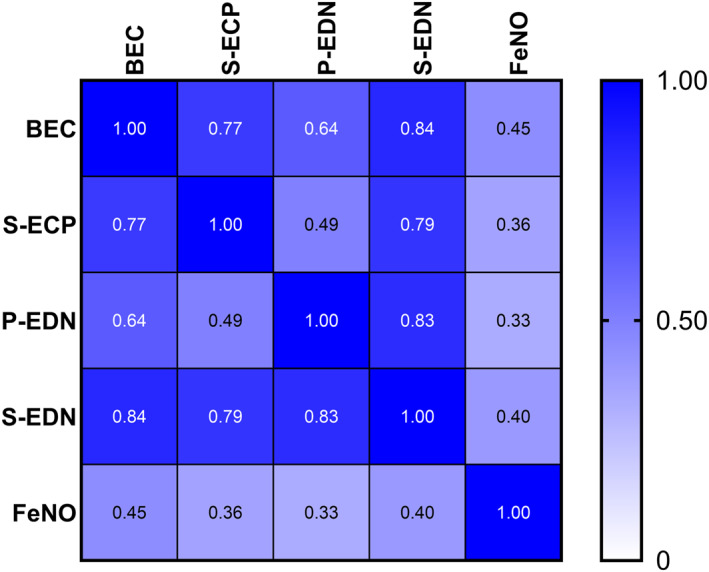
Pearson's correlation matrix between the studied biomarkers.

Eosinophil activity markers intercorrelated, with Pearson's correlation coefficients ranging between moderate (0.49; S‐ECP vs. P‐EDN) and strong (0.84; S‐EDN vs. BEC) (Figure 3). FeNO correlated weakly to moderately with eosinophil markers (0.33–0.45).

When comparing the biomarkers based on differences in AIC values (Δ*i*), S‐EDN had the lowest AIC value in models assessing the association between biomarkers and lung function (Table 5). However, for AHR and uncontrolled asthma, FeNO had the lowest AIC values, and BEC performed equally well as FeNO with regard to AHR.

**TABLE 5:** Comparison of the biomarkers' associations with lung function, AHR, and uncontrolled asthma based on AIC values and Δ*i*.

Δ*i* compared with S‐EDN (lowest)^a^	BEC	FeNO	S‐ECP	P‐EDN
FEV_1_ < 80% predicted	1.8	3.1	5.2	5.6
FEV_1_/FVC < LLN	1	1.2	5.0	3.9

Δ*i* compared with FeNO (lowest)^a^	BEC	S‐EDN	S‐ECP	P‐EDN
Moderate/severe AHR	0.1	16.7	22.5	25.9
Uncontrolled asthma	6.2	6.6	4.9	6.6

^a^ Models with Δ*i* ≤ 2 have substantial support (evidence), those with Δ*i* 4–7 have considerably less support, and models with Δ*i* > 10 have essentially no support [46].

## 4 Discussion

The present study investigated the utility of the eosinophil activity markers S‐EDN, P‐EDN, and S‐ECP, in comparison with the commonly used BEC and FeNO, to indicate risk of asthma morbidity. Our results showed that S‐EDN, but not the other activity markers, demonstrated suitability to indicate risk of reduced lung function, and could for this purpose be used as a surrogate for BEC. This finding holds particular significance given the common scenario where BEC is not available. However, FeNO and BEC were superior at indicating moderate to severe AHR, and FeNO was the only biomarker that associated with asthma control.

Out of the three eosinophilic activity markers, S‐EDN correlated most strongly with BEC, and S‐EDN exhibited a stronger association with airway obstruction and AHR than S‐ECP and P‐EDN, based on the AIC values. Furthermore, S‐EDN also seemed to associate slightly more strongly with airway obstruction than BEC and FeNO. The combination of FeNO and S‐EDN demonstrated similar results in relation to asthma outcomes as the combination of FeNO and BEC did in a previous study [31]. When assessing moderate to severe AHR, combining S‐EDN, BEC, and FeNO (aOR 9.5 [3.8–23.8]) had limited added value compared with combining FeNO and S‐EDN (7.9 [3.4–18.6]) or FeNO and BEC (aOR 10.9 [4.9–24.3]) [31]. Moreover, a combined score for all five studied biomarkers did not further strengthen the association with asthma morbidity. Further characterisation of different biomarker phenotypes could potentially enhance the translational relevance of these findings. However, given the high correlation between biomarkers, a larger study population would be required to adequately power such subgroup analyses.

Plasma measurements of EDN could be advantageous over serum measurements, as the variability during the serum preparation process would be avoided. However, we can report that S‐EDN associated more strongly with asthma outcomes than P‐EDN when strict protocols were followed during serum preparation (1 h coagulation at 21°C–23°C). This supports the view that the release of eosinophil‐derived proteins during the coagulation phase provides a clinically important signal. Levels of S‐EDN were much higher than those of P‐EDN. Interestingly, S‐EDN levels were also much higher than those of S‐ECP, even though the cellular content of EDN is lower than that of ECP [47]. This supports the view that ECP is lost both in vivo and ex vivo due to its sticking to surfaces, and S‐ECP was inferior to S‐EDN with regard to association with asthma morbidity. Previously, S‐EDN has proven to be a useful biomarker for asthma management in routine clinical practice for both children and adults [3], and our study demonstrates its superiority over P‐EDN and S‐ECP.

It is noteworthy that the cut‐off for high S‐EDN in our study, based on the 95th percentile of non‐atopic healthy controls, was lower than in a study of children at ages 1 and 3 years that used the same assay methodology [28]. However, the BEC is higher in small children than in adults [48]. The median S‐EDN value in the non‐atopic controls in our study was similar to that reported by Lee et al. [49], in a study including 125 non‐asthmatic individuals with a mean age of 51 years, and also similar to that in a report by An et al. [27], including 43 healthy controls with a mean age of 51 years. Furthermore, our reported cut‐off of 62 μg/L was similar to that reported in a Swedish general population (72.7 μg/L) [50]. It should be acknowledged that EDN levels may be influenced by factors other than asthma activity, including acute respiratory infections and allergic comorbidities such as chronic rhinosinusitis. Additionally, factors like differences in age groups, as biomarker levels vary across the lifespan, assay methodology and population selection may explain discrepancies in cut‐off values between the studies. Previous studies often included symptomatic patients from clinical settings, whereas our population‐based controls were non‐asthmatic and non‐atopic recruited randomly from the population register, which may have introduced inclusion bias, as individuals with undiagnosed respiratory problems may be more inclined to participate in such a study. The high percentage of moderate to severe AHR among the controls also indicates this. Although AHR has been reported to be at a similar level in other populations [51] and also more prevalent during childhood [52]. AHR may be influenced by recent respiratory infections or other factors. However, all subjects were told that they should have been free from symptoms of respiratory tract infections (cough, sore throat, runny nose, sneezing, nasal congestion, pink eyes or fever) for at least 2 weeks on the day of examination. These factors should be considered when comparing cut‐off values across studies.

From a clinical perspective, our findings suggest that FeNO and BEC remain the most appropriate markers for assessing AHR, and FeNO for asthma control. S‐EDN measurement may be considered in specific clinical scenarios: First, when BEC or FeNO are unavailable, such as when using stored serum samples in research settings. Second, when there is particular concern about lung function or AHR or when biomarker results are discordant or inconsistent with clinical presentation. However, it is important to note that our findings are associative rather than predictive, and the clinical utility of S‐EDN requires validation in longitudinal studies examining its ability to predict future outcomes.

A notable strength of our study lies in the comprehensive comparison of multiple biomarkers, possibly representing different aspects of eosinophilic inflammation. In addition, we had the ability to define our own cut‐offs in a well‐characterised control group from the same region, enhancing the clinical relevance of our results.

The study also has some limitations. First, the sample size of our study was relatively modest, necessitating validation of our findings in larger cohorts to enhance generalisability. Second, the observational nature of our study precludes conclusions on causal relationships. Third, our study population consisted predominantly of young patients with early‐onset, allergic asthma, and findings may differ in adult‐onset eosinophilic asthma populations where eosinophil activity may be more pronounced. Fourth, we did not explore EDN differences in relation to asthma severity, treatment adherence, previous exacerbations, or oral corticosteroid use. Fifth, the dichotomisation of biomarkers may have reduced statistical power, and future studies should explore continuous relationships. Sixth, our cut‐offs were derived from a relatively small control group (*n* = 71), and external validation against population‐based reference values is needed. Additionally, our results were obtained using a specific assay platform (ImmunoCAP), and validation using alternative assays is warranted. Further longitudinal investigations are warranted to explore the prognostic value of S‐EDN in asthma management, particularly with regard to long‐term decline in lung function.

In conclusion, our study suggests that S‐EDN may serve as a potential biomarker associated with lung function impairment in patients with asthma, while FeNO and BEC remain the primary biomarkers for assessing AHR, and FeNO for asthma control. The inclusion of S‐EDN in biomarker profiling may be particularly relevant when BEC is unavailable or when there is specific concern about lung function and AHR. Moreover, we showed that S‐EDN can be used as an alternative to BEC, for example, in a research setting. Future research should focus on corroborating our findings in larger cohorts and defining the optimal cut‐off values for S‐EDN in diverse clinical settings.

## Author Contributions

**Nils Oskar Jõgi:** writing – original draft, writing – review and editing, visualization, validation, methodology, software, formal analysis, investigation, conceptualization. **Nikolaos Tsolakis:** conceptualization, investigation, writing – review and editing, methodology, validation, data curation, formal analysis. **Anders Sjölander:** data curation, formal analysis, validation, methodology, writing – review and editing, conceptualization, investigation. **Robert Movérare:** conceptualization, investigation, funding acquisition, methodology, validation, writing – review and editing, resources, data curation, software, formal analysis. **Christer Janson:** funding acquisition, investigation, conceptualization, visualization, methodology, validation, project administration, supervision, resources, formal analysis. **Andrei Malinovschi:** funding acquisition, project administration, data curation, supervision, resources, software, formal analysis, validation, writing – review and editing, writing – original draft, investigation, conceptualization, methodology. **Kjell Alving:** funding acquisition, conceptualization, investigation, writing – original draft, project administration, data curation, supervision, resources, formal analysis, validation, writing – review and editing, methodology.

## Funding

The MIDAS study was supported within an industry‐academy collaboration framework initiated by the Swedish Governmental Agency for Innovation Systems (VINNOVA, SAMBIO Program) where Aerocrine AB (producer of exhaled NO devices), Thermo Fisher Scientific, Immunodiagnostics (producer of allergy tests) were partners and cofinanced the program with additional support from Uppsala University Hospital.

## Ethics Statement

The Uppsala Regional Ethical Review Board approved the study (approval no. 2009/349), and all subjects and their legal guardians provided written informed consent.

## Conflicts of Interest

Anders Sjölander and Robert Movérare are employed by Thermo Fisher Scientific. Kjell Alving has received research material from Thermo Fisher Scientific for this study, and has received research material from Niox Group and Hemocue outside this study. Andrei Malinovschi has received in‐kind support in form of nitric oxide sensors from NIOX (a producer of FeNO devices) within a frame of an investigator‐initiated study (not the present study). The rest of the authors declare that they have no relevant conflicts of interest. Co‐authors employed by Thermo Fisher Scientific were not involved in the data analysis.

## Data Availability

The authors confirm that all data underlying the findings are available on request from the authors.
